# Development of a multivariable prediction model to identify patients unlikely to complete a colonoscopy following an abnormal FIT test in community clinics

**DOI:** 10.1186/s12913-020-05883-2

**Published:** 2020-11-10

**Authors:** Amanda F. Petrik, Erin Keast, Eric S. Johnson, David H. Smith, Gloria D. Coronado

**Affiliations:** The Center for Health Research, Kaiser Permanente Northwest, 3800 N. Interstate Avenue, Portland, OR 97381 USA

**Keywords:** Colorectal cancer screening, Fecal immunochemical test, Colonoscopy, Multivariable prediction model, Predictive analytics, Precision medicine, Follow-up colonoscopy

## Abstract

**Background:**

Colorectal cancer (CRC) is the 3rd leading cancer killer among men and women in the US. The Strategies and Opportunities to STOP Colon Cancer in Priority Populations (STOP CRC) project aimed to increase CRC screening among patients in Federally Qualified Health Centers (FQHCs) through a mailed fecal immunochemical test (FIT) outreach program. However, rates of completion of the follow-up colonoscopy following an abnormal FIT remain low. We developed a multivariable prediction model using data available in the electronic health record to assess the probability of patients obtaining a colonoscopy following an abnormal FIT test.

**Methods:**

To assess the probability of obtaining a colonoscopy, we used Cox regression to develop a risk prediction model among a retrospective cohort of patients with an abnormal FIT result.

**Results:**

Of 1596 patients with an abnormal FIT result, 556 (34.8%) had a recorded colonoscopy within 6 months. The model shows an adequate separation of patients across risk levels for non-adherence to follow-up colonoscopy (bootstrap-corrected C-statistic > 0.63). The refined model included 8 variables: age, race, insurance, GINI income inequality, long-term anticoagulant use, receipt of a flu vaccine in the past year, frequency of missed clinic appointments, and clinic site. The probability of obtaining a follow-up colonoscopy within 6 months varied across quintiles; patients in the lowest quintile had an estimated 18% chance, whereas patients in the top quintile had a greater than 55% chance of obtaining a follow-up colonoscopy.

**Conclusions:**

Knowing who is unlikely to follow-up on an abnormal FIT test could help identify patients who need an early intervention aimed at completing a follow-up colonoscopy.

**Trial registration:**

This trial was registered at ClinicalTrials.gov (NCT01742065) on December 5, 2012. The protocol is available.

**Supplementary Information:**

The online version contains supplementary material available at 10.1186/s12913-020-05883-2.

## Background

Colorectal cancer (CRC) is the 3rd leading cancer killer in the United States. Mailed fecal immunochemical testing (FIT) outreach programs can effectively increase CRC screening rates among underserved populations [1–3]. The Strategies and Opportunities to STOP Colon Cancer in Priority Populations (STOP CRC) project aimed to increase CRC screening among patients in Federally Qualified Health Centers (FQHCs) through mailed FIT outreach [4]. The FIT looks for hidden blood in the stool, which may be a sign of polyps or cancer in the colon or rectum. An abnormal test means that blood was found in the stool. For these patients, a follow-up colonoscopy is recommended, yet rates of completion of the follow-up colonoscopy remain low [5–7]. Among patients who receive care in community clinics in the United States, follow-up colonoscopy rates are consistently as low as 50% [7–10]. Liss and Chubak have identified rates of achieving a follow-up colonoscopy after an abnormal FIT test of 54 and 50% respectively in community health center patients [11, 12]. This is of concern because delaying a follow-up colonoscopy up to 12 months following an abnormal fecal test is associated with increased cancer diagnoses and advanced cancer stage at the time of diagnosis [13].

Barriers can inhibit patients’ ability to complete this follow-up colonoscopy. Patient-level barriers to completing a follow-up colonoscopy may include fear of results, inability to take time off of work, the cost of preparation supplies or the colonoscopy, inability to complete adequate bowel prep, difficulty finding a driver on the day of the procedure, having competing health concerns, and lack of understanding that the procedure was necessary [7, 10, 14, 15].

Provider and system-level barriers may include limited colonoscopy capacity, failure to refer the patient to the specialist or schedule the procedure, failure to communicate expectations about the procedure or preparation for the procedure, and lack of adequate workflows to complete the referral [6–8]. Interventions like patient navigation, where a navigator helps address these barriers to screening, can close the gaps and improve follow-up rates [15, 16].

While it is likely cost-prohibitive to “navigate” all patients with an abnormal FIT, stratifying the patients in the greatest need of navigation could target resources to close gaps in screening. The use of the electronic health record (EHR) to identify patients at risk for failure to follow-up on abnormal screening, who may be candidates for personalized interventions, may improve the precision of healthcare delivery [17]. Therefore, we aimed to develop a multivariable prediction model using patient level data only available in the EHR to identify patients who are unlikely to undergo colonoscopy following an abnormal FIT test. We hypothesized that we could accurately predict which patients have a low probability of obtaining a colonoscopy.

Knowing who may be at risk for not adhering to recommendations for a follow-up colonoscopy after an abnormal FIT test could help providers and clinics identify patients in need of early interventions (including patient navigation) aimed at completing a colonoscopy. Precision delivery of interventions to those most likely to benefit might optimize patient outcomes and enhance opportunities to sustain successful interventions in low-resource settings.

## Methods

To predict each patient’s probability of obtaining a colonoscopy, we developed a risk prediction model using data from patients with an abnormal fecal test at the 26 STOP CRC clinics. We followed guidelines set forth by the Transparent Reporting of a multivariable prediction model for Individual Prognosis or Diagnoses statement [18, 19]. This model was designed to be put into practice at community clinics using data available in the EHR. Our objective was to predict patients who may benefit from interventions to complete the recommended follow-up.

### Setting and participants

This retrospective analysis used data from the STOP CRC project and included eligible patients who have returned a FIT with an abnormal result during the study period. OCHIN, formerly the Oregon Community Health Information Network, is a nonprofit health information technology services provider that provides a centrally hosted EHR for primary care clinics. The STOP CRC project included 26 clinics in Oregon and California that served as the setting for this cohort. This project was approved by the Kaiser Permanente Northwest IRB (Protocol #4364). Clinics operated in diverse settings were diverse in size and were part of 8 health centers.

To be eligible in the STOP CRC study, patients had to have been 50–75 years old and not up to date with CRC screening including fecal testing in the past 11 months or colonoscopy in the past 9 years. Patients were excluded from STOP CRC if they had co-morbid conditions that would make screening inappropriate, such as a history of CRC, colectomy, or dialysis. Our complete inclusion criteria are described elsewhere [20]. We then assembled a complete retrospective cohort of STOP CRC patients who subsequently completed CRC screening by FIT test and obtained an “abnormal” result. All patients with at least one abnormal FIT result from February 4, 2014, through February 28, 2016, were identified (*n* = 1723). For patients with more than one abnormal test result, the date of the most recent result was time zero, the start of follow-up.

### Outcome and duration of follow-up

The outcome measure of interest was whether a patient received a colonoscopy within 6 months of receiving their abnormal FIT test result. For the Cox model, the outcome was determined if a colonoscopy was completed within 180 days following the abnormal FIT test. Patients were not censored for loss to follow-up, as community clinics do not track membership. Completed colonoscopies were determined through procedure codes in the EHR.

### Predictor characteristics

We selected variables for our risk prediction model based on previous studies that identified predictors of failure to complete CRC screening or colonoscopy, but limited variables to those that would be available in the EHR in these community clinics (Table 1). Predictor characteristics were measured during the year before time zero unless otherwise specified. Predictors included clinic systems, patient demographics, community level characteristics, self-reported behavior (e.g., smoking history), clinical findings (e.g., body mass index, and the number of missed appointments), medications (e.g., antihypertensive medications), and diagnoses (e.g., history of cardiovascular disease). All coding and measurement of variables are described in the Additional file 1. Community data variables were collected at the Census tract level for all variables except for emergency department (ED) visits per 1000 enrollees; this was collected at the county level. Community-level variables were obtained from the ADVANCE Clinical Data Research Network, which is a data-source integrated into the OCHIN data [21].

**Table 1:** Characteristics at baseline for all patients and patients with a colonoscopy

	All Patients		With Colonoscopy	Univariate		Likelihood Ratio
	N	(% of all)	N	(% row)	HR	*p*-value
All	1596	100.00%	556	34.80%		
***Age***						0.0040
***50–54***	498	31.20%	200	40.20%	ref	
***55–59***	425	26.60%	156	36.70%	0.91	
***60–64***	377	23.60%	122	32.40%	0.76	
***65–69***	202	12.70%	62	30.70%	0.71	
***70–75***	94	5.90%	16	17.00%	0.37	
Sex						0.4032
Male	757	47.40%	275	36.30%	ref	
Female	839	52.60%	281	33.50%	0.9	
BMI						0.1467
< 24	420	26.30%	143	34.10%	ref	
25–29	453	28.40%	149	32.90%	0.98	
30–34	349	21.90%	137	39.30%	1.22	
35–39	209	13.10%	65	31.10%	0.89	
40+	165	10.30%	62	37.60%	1.15	
Language						0.0780
Non-English	312	19.60%	95	30.50%	ref	
English	1284	80.50%	461	35.90%	1.26	
***Race***						0.0599
***Non-White***	266	16.70%	72	27.10%	ref	
***White***	1330	83.30%	484	36.40%	1.48	
Ethnicity						0.0270
Non-Hispanic	1445	90.50%	496	34.30%	ref	
Hispanic	151	9.50%	60	39.70%	1.21	
***Insurance***						0.5652
***Uninsured***	265	16.60%	86	32.50%	ref	
***Medicaid***	748	46.90%	282	37.70%	1.18	
***Medicare***	435	27.30%	136	31.30%	0.94	
***Commercial***	148	9.30%	52	35.10%	1.1	
Tobacco Use						0.9812
Never/Quit	1153	72.20%	393	34.10%	ref	
Current User	443	27.80%	163	36.80%	1.11	
Percent of Census Tract with College Degree						0.0697
4.9–14.6	346	21.70%	125	36.10%	ref	
14.7–19.9	337	21.10%	96	28.50%	0.73	
19.9–25.7	324	20.30%	125	38.60%	1.08	
26.0–36.8	282	17.70%	94	33.30%	0.88	
36.9–77.7	307	19.20%	116	37.80%	1.04	
Percent of Census Tract Households below FPL						0.2315
2.7–11.4	283	17.70%	105	37.10%	ref	
11.4–14.8	288	18.10%	100	34.70%	0.91	
14.9–19.4	309	19.40%	105	34.00%	0.89	
19.5–25.8	333	20.90%	131	39.30%	1.05	
26.1–53.9	383	24.00%	115	30.00%	0.75	
Census Tract Median Household Income						0.6530
$14,000 - $36,000	331	20.70%	101	30.50%	ref	
$36,000 - $41,000	330	20.70%	125	37.90%	1.32	
$41,000 - $47,000	353	22.10%	117	33.10%	1.11	
$47,000 - $56,000	286	17.90%	101	35.30%	1.18	
$56,000 - $149,000	296	18.60%	112	37.80%	1.33	
Census Tract Unemployment						0.0009
2.6–8.1	323	20.20%	132	40.90%	ref	
8.2–10.2	285	17.90%	83	29.10%	0.65	
10.2–12.7	293	18.40%	88	30.00%	0.68	
12.7–15	397	24.90%	146	36.80%	0.87	
15.0–32.4	298	18.70%	107	35.90%	0.84	
Census Tract Population Density (People per square mile of land area)						0.2521
0.8–174	238	14.90%	96	40.30%	ref	
176–1571	217	13.60%	62	28.60%	0.67	
1574 - 3770	289	18.10%	83	28.70%	0.64	
3781 - 6576	358	22.40%	144	40.20%	0.98	
6593 - 26,873	494	31.00%	171	34.60%	0.8	
***Census Tract GINI Income Inequality***						0.4162
***0.27–0.38***	329	20.60%	102	31.00%	ref	
***0.38–0.41***	326	20.40%	115	35.30%	1.15	
***0.41–0.43***	326	20.40%	122	37.40%	1.24	
***0.43–0.47***	256	16.00%	97	37.90%	1.26	
***0.47–0.82***	359	22.50%	120	33.40%	1.09	
Low access Census Tract at 1/2 mile for urban areas or 5 miles for rural areas						0.8152
No	309	19.40%	121	39.20%	ref	
Yes	1287	80.60%	435	33.80%	0.81	
Emergency Room Visits per 1000 Medicare Enrollees (County)						0.7264
0	356	22.30%	107	30.10%	ref	
1	914	57.30%	337	36.90%	1.24	
2+	326	20.40%	112	34.40%	1.16	
Urban/Rural County						0.7809
Cluster (10-50 k population)	276	17.30%	84	30.40%	ref	
Rural (< 10 K population)	246	15.40%	97	39.40%	1.39	
Urban (50 k + population)	1074	67.30%	375	34.90%	1.16	
Charlson Comorbidity						0.7870
0	705	44.20%	259	36.70%	ref	
1	465	29.10%	159	34.20%	0.94	
2	213	13.40%	71	33.30%	0.89	
3+	213	13.40%	67	31.50%	0.83	
Asthma/COPD dx in 2 years prior to index						0.1816
No	1122	70.30%	404	36.00%	ref	
Yes	474	29.70%	152	32.10%	0.87	
Diabetes dx in 2 years prior to index						0.2072
No	881	55.20%	322	36.60%	ref	
Yes	715	44.80%	234	32.70%	0.86	
Severe mental illness						0.7889
No	1455	91.20%	504	34.60%	ref	
Yes	141	8.80%	52	36.90%	1.09	
Mood disorder (Depression, Bipolar) dx in 2 years prior to index						0.6492
No	1006	63.00%	342	34.00%	ref	
Yes	590	37.00%	214	36.30%	1.1	
Substance/alcohol abuse dx in 2 years prior to index						0.6928
No	1264	79.20%	434	34.30%	ref	
Yes	332	20.80%	122	36.80%	1.14	
***Long term anticoagulant use***						0.0353
***No***	1545	96.80%	546	35.30%	ref	
***Yes***	51	3.20%	10	19.60%	0.5	
Blood in Stool prior to abnormal FIT						0.3026
No	1538	96.40%	538	35.00%	ref	
Yes	58	3.60%	18	31.00%	0.86	
Hemorrhoid/Anal Fissure prior to abnormal FIT						0.3546
No	1514	94.90%	526	34.70%	ref	
Yes	82	5.10%	30	36.60%	1.08	
Prior CRC screening						0.2966
No	985	61.70%	362	36.80%	ref	
Yes	611	38.30%	194	31.80%	0.82	
***Flu shot within 1 year of index date***						0.0000
***No***	1368	85.70%	452	33.00%	ref	
***Yes***	228	14.30%	104	45.60%	1.57	
Number of outpatient encounters in the year prior to index date						0.3248
0	203	12.70%	85	41.90%	ref	
1	173	10.80%	52	30.10%	0.65	
2	196	12.30%	59	30.10%	0.64	
3	209	13.10%	80	38.30%	0.86	
4	147	9.20%	50	34.00%	0.73	
5	119	7.50%	42	35.30%	0.79	
6+	549	34.40%	188	34.20%	0.77	
***Count of no-show encounters in the year prior to index date***						0.0022
***0***	1128	70.70%	394	34.90%	ref	
***1***	253	15.90%	99	39.10%	1.16	
***2+***	215	13.50%	63	29.30%	0.82	
***Health Center***						0.0000
***HC 8***	155	9.70%	31	20.00%	ref	
***HC 7***	70	4.40%	19	27.10%	1.45	
***HC 4***	104	6.50%	44	42.30%	2.57	
***HC 2***	615	38.50%	193	31.40%	1.62	
***HC 5***	287	18.00%	139	48.40%	3.03	
***HC 6***	232	14.50%	66	28.50%	1.43	
***HC 3***	133	8.30%	64	48.10%	3.12	

### Statistical analysis

We evaluated the characteristics predicting follow-up colonoscopy using a Cox proportional hazards model and a logistic model in SAS® System Software. We fit a full model of patients with complete data and used a step-down process to manually remove the weakest characteristics one covariate at a time to simplify the model so that the final model retained at least 90% of the variation explained of the full model.

For the final model, we calculated the mean observed risk of completing the colonoscopy and plotted mean observed and predicted risks in quintiles using risk predictiveness curves that showed the distribution of predicted risks of completing the colonoscopy [22]. Discrimination was measured by a bootstrap corrected C-statistic. Variation explained was measured with an R^2^ statistic. The Cox regression coefficients were then translated into a simplified point-based risk scoring system to improve use in the clinical setting. A higher number of points mean a higher likelihood of completing a follow-up colonoscopy [23]. This allows clinicians to translate the model into practice without calculating the regression equation exactly. Table 2 shows the expected and observed probability of completing a colonoscopy within 6 months of an abnormal FIT test by points. The points assignment reflects the variations in hazard ratios across patient characteristics. The clinician could add up the points to determine likelihood of completing the follow-up colonoscopy.

**Table 2:** Expected and observed probability of completing a colonoscopy within 6 months of abnormal FIT test, by points score

Points Score^**a**^	Expected Probability, %	Observed probability, %	(95% CI)
140–149	17.30%	18.00%	(7.0–29.0)
150–159	19.20%	14.50%	(6.4–22.6)
160–169	21.30%	18.10%	(10.2–26.0)
170–179	23.60%	27.90%	(19.8–35.9)
180–189	26.10%	23.60%	(16.1–31.1)
190–199	28.80%	28.80%	(21.5–36.0)
200–209	31.80%	33.80%	(25.9–41.8)
210–219	34.90%	40.70%	(32.3–49.1)
220–229	38.30%	34.60%	(26.4–42.8)
230–239	41.90%	39.60%	(30.2–49.1)
240–249	45.70%	45.20%	(34.9–55.5)
250–259	49.60%	54.70%	(43.1–66.2)
260–269	53.70%	54.30%	(44.0–64.5)
270–279	57.90%	46.40%	(33.0–59.9)
280–289	62.20%	66.70%	(51.2–82.1)
290–299	66.50%	58.30%	(37.1–79.6)

^a^Probabilities calculated for point ranges with at least 20 patients

## Results

**Fig. 1:**
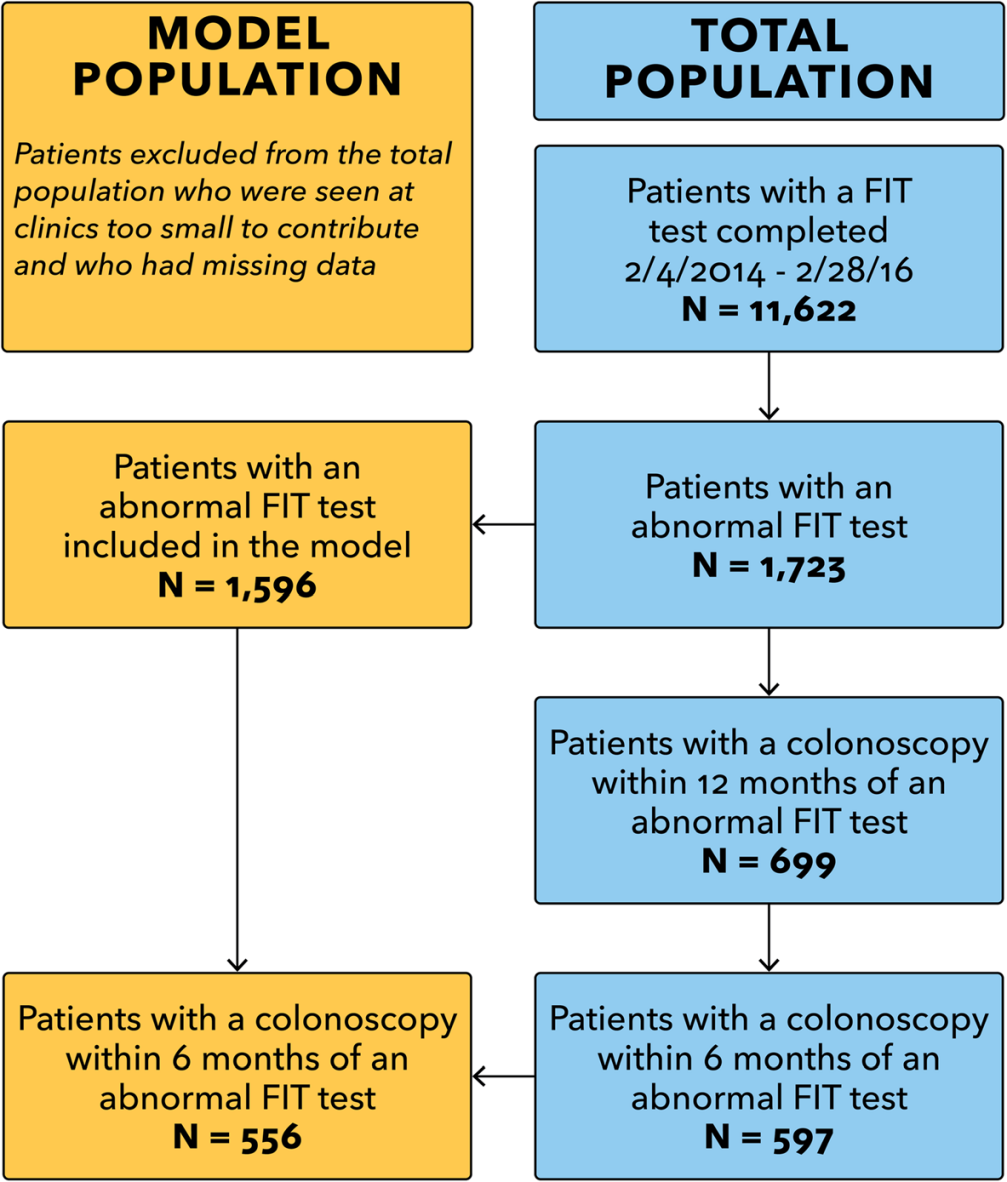
Risk Model and Patient Population from STOP CRC

Of 11,622 patients with a completed fecal test, 1723 (14.8%) were abnormal, and 699 (40.6%) of those had a subsequently completed colonoscopy in their EHR record within 12 months (Fig. 1). However, only 597 (34.6%) of those patients had a record of a completed a colonoscopy within 6 months of their abnormal FIT test. For this analysis, one small clinic system was excluded due to low numbers of patients with abnormal FIT results (*n* = 13). We also only included patients with non-missing data for all predictors (*n* = 1596). Of the 1596 patients included in the final model, 34.8% (*n* = 556) had recorded completed colonoscopies within 6 months.

Table 1 illustrates all baseline characteristics for the entire cohort and the subgroup that had a recorded completed colonoscopy within 6 months. Overall, patients were typically white (83.3%), aged 50–64 (81.5%), and had a low rate of preventive screenings: flu shots (14.3%); prior CRC screening (38.3%)). Only eight variables were retained for the final model as they contributed to the explained variation in risk.

The eight characteristics retained in the final Cox regression model included age, race, insurance, GINI income inequality, long term anticoagulant use, receipt of a flu vaccine in the past year, frequency of missed clinic appointments, and health center (Table 3). No notable differences were determined when the model was run for men and women separately, so therefore we combined men and women to develop one model. Table 3 also shows hazard ratios, confidence intervals, and the number of risk points assigned to each characteristic. The hazard ratios and risk score points for the final prediction model indicated that health center, age, long term anticoagulant use, and receipt of a flu vaccine in the past year were the variables with the highest points assigned in the model.

**Table 3:** Hazard ratios and risk score points for the final prediction model

Variable	Hazard Ratio	(95% CI)	Likelihood ratio p-value	Points
Age			0.0011	
50–54	ref			83
55–59	0.92	(0.74–1.13)		76
60–64	0.76	(0.61–0.96)		60
65–69	0.76	(0.55–1.04)		59
70–75	0.38	(0.22–0.65)		0
Race			0.0019	
Non-White	ref			0
White	1.48	(1.14–1.91)		34
Insurance			0.5174	
Uninsured	ref			3
Medicaid	1.15	(0.90–1.48)		15
Medicare	1.03	(0.77–1.38)		5
Commercial	0.97	(0.67–1.40)		0
Census Tract GINI Income Inequality			0.4446	
0.27–0.38	ref			0
0.38–0.41	1.14	(0.87–1.49)		11
0.41–0.43	1.17	(0.90–1.53)		14
0.43–0.47	1.25	(0.94–1.66)		19
0.47–0.82	1.27	(0.97–1.67)		21
Long term anticoagulant use			0.0315	
No	ref			54
Yes	0.54	(0.29–1.01)		0
Flu shot within 1 year of index date			0.0001	
No	ref			0
Yes	1.59	(1.28–1.98)		40
Count of no-show encounters in the year prior to the index date			0.0151	
0	ref			31
1	1.07	(0.86–1.34)		37
2+	0.7	(0.53–0.92)		0
Health Center			0.0000	
HC 8	ref			0
HC 7	1.45	(0.82–2.58)		32
HC 4	2.59	(1.62–4.14)		82
HC 2	1.65	(1.12–2.44)		43
HC 5	3.01	(2.02–4.49)		95
HC 6	1.33	(0.86–2.06)		25
HC 3	3.18	(2.05–4.92)		100

**Fig. 2:**
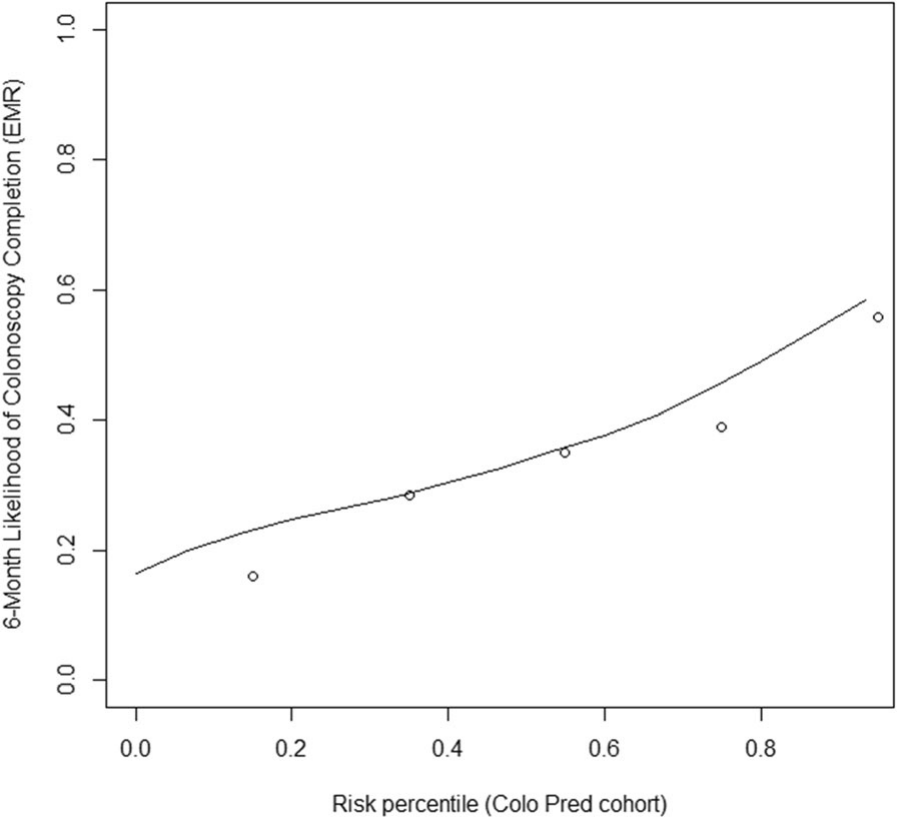
Observed and Predicted Probability Colonoscopy Completion

The mean predicted risk of completion of colonoscopy was 34.8%, and the model was able to accurately predict the patients who were least likely to receive a follow-up colonoscopy (lowest two quintiles, 15.9, and 28.5% respectively). The likelihood of obtaining a follow-up colonoscopy within 6 months varied across quintiles: patients with the highest predicted risk of non-adherence (bottom quintile) had an estimated 16% chance of obtaining a colonoscopy; whereas, patients with the lowest predicted risk of non-adherence (top quintile) had a greater than 55% chance of obtaining a follow-up colonoscopy. Figure 2 shows the predictiveness curve for colonoscopy completion. The open circles are the observed proportions (o) and the line represents the predicted probability of colonoscopy completion.

Risk score points can be assigned to a patient to determine their risk of completing a colonoscopy. For example, we can score a patient who is on Medicaid (15 points), white (34 points), 54 years old (83 points), receives his care at health center 3 (100 points), has not missed appointments (31 points), has received a flu shot (40 points), isn’t on anticoagulants (54 points) and lives in an area with low-income inequality (21 points). His total point count is 378, which predicts that he has an 81% probability of completing a colonoscopy, compared to the 35% likelihood of the average patient (data not shown).

The model showed modest separation of patients across risk levels for non-adherence to follow-up colonoscopy (C-statistic> 0.66, bootstrap-corrected C-statistic> 0.63) and excellent calibration or high agreement between observed and predicted risk. The R^2^ statistic, derived from the D-statistic, showed only 14% of the variation in outcome was explained in this model (R^2^ (95% CI) ^=^14.03 (10.17–18.18), D (95% CI) = 0.83 (0.69–0.96)). A logistic regression, predicting the completion of a colonoscopy, showed similar results for non-adherence to follow-up colonoscopy (C-statistic = 0.66, bootstrap-corrected C-statistic> 0.64).

## Discussion

This model was created to identify patients at the greatest need for targeted interventions, such as patient navigation, to complete the screening process for CRC. We recognize that the performance of the model has limitations. The C-statistic, while suboptimal, shows the adequate separation of patients across risk levels for non-adherence to follow-up colonoscopy, yet the R^2^ indicates the discrimination and calibration could be further improved. However, focusing efforts on improving follow-up colonoscopy among patients in the lowest quintiles could provide value in the population most in need of understanding the importance of follow-up. Identifying the barriers among these patients and targeting interventions could produce improvements. When putting the model into practice, targeting the lowest probability groups could result in the greatest improvements.

Both patient and system level barriers were used in the final model, indicating the importance of recognizing multilevel barriers in adherence to colonoscopy following an abnormal FIT. The strongest predictors were age, health center, anticoagulant use, and flu shot vaccination. The youngest patients were more likely to obtain colonoscopies, which may be explained by newer entry into screening eligibility and they are healthier. However, recognizing a lower likelihood of follow-up screening among older patients could help in efforts to close the gap in colonoscopy completion. Patients’ likelihood of receiving colonoscopy varied by the health center, and this is a complex variable that could represent a variety of systems and patient level factors. While the system level factors impacting colonoscopy completion can include access to colonoscopies, location, and community characteristics, the referral process, scheduling, waitlists, and capacity, it is also affected by many patient level barriers reflected in the health system like transportation barriers, inability to take time off work and mistrust of the system. The health system variable is important in this model and should be further examined.

Patients indicated as users of anticoagulant medications were less likely to obtain a colonoscopy following an abnormal FIT. Removing a patient from an anticoagulant may be required before a colonoscopy and could be a deterrent for completing the test. Patients who were vaccinated for the flu in the past year were more likely to complete colonoscopy, indicating a trend of compliance for recommended preventative care. Patients with Medicaid insurance, a free or low-cost health coverage for low-income patients, were more likely to complete the colonoscopy. This may indicate program effectiveness or the effect of removing cost barriers. The GINI Income Inequality Ratio is a statistical measure of income inequality where a measure of 1 indicates total inequality and a measure of 0 indicates total equality. This analysis shows that living in an area of inequity (closer to 1.0) decreases one’s chances of completing a colonoscopy. Finally, patients who were non-white were less likely to complete a colonoscopy. Addressing issues among non-white populations through interventions is necessary to close the gap in disparities in CRC screening.

We believe this is the first model to predict the likelihood of follow-up after an abnormal fecal test. Further research is needed to test the effectiveness of interventions for patients who have a low and moderate probability of completing follow-up colonoscopy.

### Limitations

There are known deficiencies in capturing completed colonoscopies and referrals in the EHR, and especially in the community clinic setting where most colonoscopies are referred to outside providers or specialties without a direct link to the EHR. Therefore, updating records relies on clinic processes. While analyzing chart abstracted colonoscopies could be the gold standard, it only explains why patients may or may not have colonoscopies recognized by the physician in obscure data points in the EHR [24]. This population is a primarily FQHC population and is therefore not generalizable to patients who obtain care in other types of clinics or healthcare settings. Further, this population is primarily in Oregon and Northern California, indicating regional limitations to generalization. Other populations and settings may not have EHR records to capture the predictors in our model. The health center variable is highly collinear with the other variables except for age. System-level predictors may matter more than patient-level predictors and these models may need to be validated for each population and setting where they will be put into practice. We sought to develop a model that will transport to other health systems, clinics, and populations. The validity of the analysis would be increased with external validation and could support widespread use.

## Conclusions

Understanding the differences in patients who are more likely to complete colonoscopy, can lead to tailored outreach to patients in need of interventions. Doing so will target resources, reduce disparities, and save lives.

## Supplementary Information

**Additional file 1:** **Appendix Table 1.** Initial list of Predictors.

## Data Availability

The datasets used and/or analyzed during the current study are not currently publicly available, however, can be made available by reasonable request to the corresponding author.

## Abbreviations

CRC: Colorectal cancer
ED: Emergency department
EHR: Electronic health record
FIT: Fecal immunochemical test
FQHC: Federally Qualified Health Center
STOP CRC: Strategies and Opportunities to STOP Colon Cancer in Priority Populations
