# Supplementary material for: Development of a multivariable prediction model to identify patients unlikely to complete a colonoscopy following an abnormal FIT test in community clinics
Source: BMC Health Serv Res. 2020 Nov 10;20:1028. doi: 10.1186/s12913-020-05883-2 (PMC7654150; doi:10.1186/s12913-020-05883-2)
Supplement: Supplementary file 1 — Additional file 1: Appendix Table 1. Initial list of Predictors. [file 12913_2020_5883_MOESM1_ESM.doc]

**Appendix Table 1:** Initial list of Predictors

| **Predictor** | **Measurement** | **Data Origin** |
| --- | --- | --- |
| Age | 5 year age bands | EHR |
| Gender | Male/Female | EHR |
| BMI | 5-unit bands (<20-25, 25-29, 30-34, 35-39, 40+) | Most recent prior, EHR |
| Language | English vs Other exclude unknown) | EHR |
| Race | White (includes unknown)/Non-white | EHR |
| Ethnicity | Hispanic, Not-Hispanic | EHR |
| Insurance | Medicaid, Medicare, Commercial (mutually exclusive, in that order) | EHR |
| Tobacco Use | Current vs never/quit/unknown | EHR |
| College Graduate | Percent of persons with a Bachelors degree or higher | American Community Survey |
| Median Household Income | Median household income | US Census Bureau |
| Unemployment Rate | Rate of unemployment | American Community Survey |
| GINI Income Inequality Ratio | With a value ranging from 0-1, this is a statistical measure of income inequality. A measure of 1 indicates total inequality and a measure of 0 indicates total equality. | American Community Survey |
| Urban/Rural | Urban and rural classification. Urban area is classified as having a population of greater than 50,000; Urban clusters of at least 2,500 and less than 50,000 and rural as all population, housing and territory not included within an urban area | US Census Bureau |
| Population Density | Number of people per square mile of land area. Calculated by dividing the total U.S. population by the total U.S. land area. | American Community Survey |
| 1 mile access | County level data on the percent of people living more than 1 mile from a supermarket or large grocery store (if living in an urban area), or more than 10 miles (if in a rural area) | USDA Food Atlas |
| Emergency Department Visits | Emergency department visits per 1,000 Medicare or Medicaid enrollees | Centers for Medicare and Medicaid |
| Household Poverty | Percentage of households with income below the federal poverty line | US Census Bureau |
| Charlson Comorbidity Index | Count (0-3+) | In prior year, EHR |
| Asthma/COPD | 490, 491.0, 491.1, 491.2, 491.20, 491.21, 491.22, 491.8, 491.9, 492.0, 492.8, 493.00, 493.01, 493.02, 493.10, 493.11, 493.12, 493.20, 493.21, 493.22, 493.81, 493.82, 493.90, 493.91, 493.92, 494, 494.0, 494.1, 496 | In prior two years, EHR |
| Diabetes | 249.00, 249.01, 249.10, 249.11, 249.20, 249.21, 249.30, 249.31, 249.40, 249.41, 249.50, 249.51, 249.60, 249.61, 249.70, 249.71, 249.80, 249.81, 249.90, 249.91, 250.00, 250.01, 250.02, 250.03, 250.10, 250.11, 250.12, 250.13, 250.20, 250.21, 250.22, 250.23, 250.30, 250.31, 250.32, 250.33, 250.40, 250.41, 250.42, 250.43, 250.50, 250.51, 250.52, 250.53, 250.60, 250.61, 250.62, 250.63, 250.70, 250.71, 250.72, 250.73, 250.80, 250.81, 250.82, 250.83, 250.90, 250.91, 250.92, 250.93, 790.2, 790.21, 790.22, 790.29, 791.5, 791.6, V45.85, V53.91, V65.46 | In prior two years, EHR |
| Severe mental health | 2 or more diagnoses of Bipolar Spectrum Disorder, PTSD, Schizophrenia, or Psychosis  295.00, 295.01, 295.02, 295.03, 295.04, 295.05, 295.10, 295.11, 295.12, 295.13, 295.14, 295.15, 295.20, 295.21, 295.22, 295.23, 295.24, 295.25, 295.30, 295.31, 295.32, 295.33, 295.34, 295.35, 295.40, 295.41, 295.42, 295.43, 295.44, 295.45, 295.50, 295.51, 295.52, 295.53, 295.54, 295.55, 295.60, 295.61, 295.62, 295.63, 295.64, 295.65, 295.70, 295.71, 295.72, 295.73, 295.74, 295.75, 295.80, 295.81, 295.82, 295.83, 295.84, 295.85, 295.90, 295.91, 295.92, 295.93, 295.94, 295.95, 296.10, 296.11, 296.12, 296.13, 296.14, 296.15, 296.16, 296.40, 296.41, 296.42, 296.43, 296.44, 296.45, 296.46, 296.50, 296.51, 296.52, 296.53, 296.54, 296.55, 296.56, 296.60, 296.61, 296.62, 296.63, 296.64, 296.65, 296.66, 296.7, 296.80, 296.81, 296.89, 297.1, 297.3, 298.8, 298.9, 301.22, 309.81 | In prior two years, EHR |
| Mood Disorder | 293.83, 296.00, 296.01, 296.02, 296.03, 296.04, 296.05, 296.06, 296.10, 296.11, 296.12, 296.13, 296.14, 296.15, 296.16, 296.20, 296.21, 296.22, 296.23, 296.24, 296.25, 296.26, 296.30, 296.31, 296.32, 296.33, 296.34, 296.35, 296.36, 296.40, 296.41, 296.42, 296.43, 296.44, 296.45, 296.46, 296.50, 296.51, 296.52, 296.53, 296.54, 296.55, 296.56, 296.60, 296.61, 296.62, 296.63, 296.64, 296.65, 296.66, 296.7, 296.80, 296.81, 296.82, 296.89, 296.90, 296.99, 300.4, 311 | In prior two years, EHR |
| Substance abuse | 291.0, 291.1, 291.2, 291.3, 291.4, 291.5, 291.8, 291.81, 291.82, 291.89, 291.9, 292.0, 292.11, 292.12, 292.2, 292.81, 292.82, 292.83, 292.84, 292.85, 292.89, 292.9, 303.00, 303.01, 303.02, 303.03, 303.90, 303.91, 303.92, 303.93, 304.00, 304.01, 304.02, 304.03, 304.10, 304.11, 304.12, 304.13, 304.20, 304.21, 304.22, 304.23, 304.30, 304.31, 304.32, 304.33, 304.40, 304.41, 304.42, 304.43, 304.50, 304.51, 304.52, 304.53, 304.60, 304.61, 304.62, 304.63, 304.70, 304.71, 304.72, 304.73, 304.80, 304.81, 304.82, 304.83, 304.90, 304.91, 304.92, 304.93, 305.00, 305.01, 305.02, 305.03, 305.20, 305.21, 305.22, 305.23, 305.30, 305.31, 305.32, 305.33, 305.40, 305.41, 305.42, 305.43, 305.50, 305.51, 305.52, 305.53, 305.60, 305.61, 305.62, 305.63, 305.70, 305.71, 305.72, 305.73, 305.80, 305.81, 305.82, 305.83, 305.90, 305.91, 305.92, 305.93, 357.5, 425.5, 535.30, 535.31, 571.0, 571.1, 571.2, 571.3, 648.30, 648.31, 648.32, 648.33, 648.34, 655.50, 655.51, 655.53, 760.71, 760.72, 760.73, 760.75, 779.5, 965.00, 965.01, 965.02, 965.09, 980.0, V65.42 | In prior two years, EHR |
| Use of anticoagulant medication | V58.61 (Long-term use of anticoagulants) | In prior year, EHR |
| Hemorrhoid/anal fissure | 455%, 565.0 | In prior two years, EHR |
| Blood in stool | 578.1 | In prior two years, EHR |
| Prior CRC screening | Prior screening by either FIT or colonoscopy | Ever in patient history, EHR |
| Flu Shot | 1 year | In prior year, EHR |
| Visits in the past year | 0-6+ | In prior year, EHR |
| No-Shows | 0,1,2+ | In prior year, EHR |
| Health Center | STOP CRC Assigned clinic | EHR |
